# Supplementary material for: Are carbon emissions trading and green financial instruments synergistic? -Comprehensive quantitative research based on content analysis
Source: PLoS One. 2024 Mar 7;19(3):e0298601. doi: 10.1371/journal.pone.0298601 (PMC10919601; doi:10.1371/journal.pone.0298601)
Supplement: S1 Data — (ZIP) [file pone.0298601.s001.zip › Data/地级市Z_F工作报告.zip!/╔┬╬≈/▒a╝a╩╨/└┤╫╘╣1⁄2╓┌║┼í╛┬φ┐╦╩2╛▌═°í┐_2013.docx]

各位代表：

现在，我代表市人民政府向大会作工作报告，请予审议，并请市政协委员和其他列席人员提出意见。

2012年工作完成情况

过去的一年，在市委的正确领导下，新一届政府认真贯彻中、省一系列决策部署，紧紧围绕“打造关天副中心、建设和谐新宝鸡，实现六个走在前列”的奋斗目标，创新进取，真抓实干，圆满完成了市十四届人大一次会议确定的各项目标任务，列入政府工作报告的299项任务，除编制旅游业发展战略规划一项予以调整外，其余全部完成了预期目标。全年实现地区生产总值1409.87亿元，比上年增长15.1%；财政总收入达到159.28亿元，增长16%，其中地方财政收入64.85亿元，增长26.1%；城镇居民人均可支配收入、农村居民人均纯收入分别达到25777元、7373元，增长15.4%和16.3%，主要经济指标增幅位居全省前列。

招商引资实现重大突破，项目建设全面提速。坚持把招商引资作为“一号工程”、“一把手工程”，突出优势资源招商、主要领导招商、优化环境招商，策划组织了45次招商活动，市县区主要领导带队招商103批次，成功引进了新希望、联想等国内知名企业，全年落实招商引资项目944个，到位资金533.69亿元，居全省前列。“央企进陕”、“民企进陕”、“省企进宝”活动扎实开展，签约项目106个，总投资920亿元。严格落实项目建设“五个一”工作机制，强力推进签约项目抓开工、在建项目抓进度、竣工项目抓达产，全市100个重点项目完成投资475.3亿元，超出年度计划7.6个百分点。全年完成全社会固定资产投资1311.69亿元，增长30.1%。支持大企业在市域内扩张，宝钛工业园钛材加工、西凤酒灌装中心、宝石机械搬迁、宝成航空精密制造基地、农夫山泉一期等41个产业项目建成投产，郭家河矿井项目获得国家核准，150万吨煤制甲醇一期、国核锆材生产线等项目进展顺利，青啤宝鸡工业园、太白钨钼丝棒材、陇县阳光凯迪、凤县新能源等项目开工建设。重大交通项目实现新突破，宝汉高速、连霍高速市区过境段、绛法高速、宝兰客专开工建设，西宝客专、宝麟铁路建设进展顺利，西宝北线、中线拓宽改造工程路基基本贯通。围绕关天副中心城市建设，超前谋划储备了740个重大项目，计划总投资7000亿元；争取中省各类项目资金130亿元，创历史新高。

工业经济逆势上扬，创新能力显著提升。把加快工业发展作为稳增长的首要任务，强化要素保障、政策支持、服务跟进，市财政安排1.4亿元专项资金，扶持工业发展，工业经济的速度、质量和效益明显提升。紧紧围绕九大产业集群，实施了百户配套企业成长计划和中小企业成长工程，全年新增规模以上工业企业64户，规模以上工业实现增加值690.61亿元，增长22.6%，高于全省平均增速6个百分点，较上年加快了3.3个百分点。骨干企业引领作用进一步增强，12户大企业大集团累计完成工业总产值983.02亿元，占规模以上工业的50.8%。秦川机床、宝石机械、中铁宝桥、法士特集团荣获中国装备制造行业排头兵企业。技术创新取得新成效，国家科技成果转化服务示范基地获批建设，国家知识产权示范城市通过验收，成为全省首批促进科技和金融结合试点市，新增省级企业技术中心5个。高新区实现生产总值450亿元，增长16%，在国家高新区综合排名中前进到第36位，被评为国家高新区开发建设二十年先进集体。高新区钛材及深加工、蔡家坡经开区专用车及零部件被批准为国家新型工业化产业示范基地。11个重点县域工业园完成固定资产投资66亿元，新增入园企业72户，凤翔长青、渭滨姜谭入选全省十大工业园区。组建了市国有资产经营公司，完成了原宝鸡酒精厂、叉车五厂改制重组。出台了加快建筑业改革与发展的意见，新增资质以上建筑企业21户，建筑业实现增加值161.58亿元，增长16.3%。

农业基础更加稳固，发展活力不断增强。坚持以增加农民收入为核心，推动“三农”工作稳步发展。全年粮食总产153.59万吨，实现了“九连丰”。新建苹果园11万亩、猕猴桃园4万亩，新栽核桃13.8万亩、花椒2.1万亩，新增蔬菜种植面积4.15万亩，总面积分别达到102.2万亩、50.3万亩、127万亩、88万亩和85.4万亩，百万亩苹果、百万亩核桃产业带初步形成。全市奶牛、生猪、肉牛、羊、家禽存栏分别达到20.76万头、110.65万头、29.24万头、53.14万只、950.46万只；现代牧业二期项目、10个标准化奶牛小区建成投用，3个千头牛场、凤县红光10万头生猪育肥场建设进展顺利。现代农业园区建设成效明显，新增省级园区11个，累计达到15个；宝鸡休闲农业示范区被命名为全国休闲农业与乡村旅游示范区，创建省级休闲农业示范点3个。农业产业化水平持续提升，新增省级以上重点龙头企业22户，总数达到49户；创建国家级农民专业合作示范社4个、省级百强示范社25个，被农业部确定为“农社对接”试点市，眉县国家级猕猴桃批发交易中心开工建设。水利基础设施不断改善，新增有效灌溉面积3万亩，新建基本农田7.81万亩；新建渭河堤防88.43公里，实现堤防堆筑基本贯通，渭河综合整治走在全省前列；20座小型水库除险加固主体工程、金陵河等5条中小河流治理项目全面完成，石头河引水一期工程、冯家山水库灌区更新改造进展顺利；建成农村集中供水工程210处，解决了23万人安全饮水问题；国家节水型社会创建工作扎实开展，33项考核指标基本达标。“绿色宝鸡”建设扎实推进，新建绿色长廊318公里，完成造林绿化46.2万亩、4220万株，建成渭河、北坡义务植树基地7800亩。陇县、麟游、千阳、扶风和太白分别纳入国家连片特困地区六盘山片区和秦巴山片区，制定并启动实施区域发展与扶贫攻坚规划。突破西山持续推进，全市建设“三无户”安全住房1000户，移民示范村58个，搬迁贫困群众9656人，实现脱贫74650人。农村环境综合整治深入开展，眉县、陈仓等9个县区列入全省连片整治示范区域，农村环境更加优美。

文化旅游引领作用凸显，第三产业稳步发展。精品旅游景区建设扎实推进，宝鸡旅游目的地影响力进一步增强。全年接待国内外游客2750万人次，实现旅游总收入176亿元，分别增长31%和33%。法门寺5A级旅游景区创建通过省级验收，太白山国家级旅游产业新区建设进展顺利；茵香河文化旅游区域综合开发加快推进，《炎黄大帝》外景拍摄基地顺利竣工，中华石鼓园工程荣获“鲁班奖”。金台观道文化景区一期、宝鸡民俗博物馆、千湖国家湿地公园、东岭石鼓文化廊桥建成开放。成功实施了石鼓山西周墓葬考古发掘，“赫赫宗周—西周文化特展”在台湾引起轰动，唐九成宫遗址保护大纲规划通过国家评审。商贸物流业加快发展，新增限额以上商贸企业125户，全年实现社会消费品零售总额409.93亿元，增长15.6%，实现外贸进出口总值7.45亿美元。工业品市场、万福七星国际酒店等一批重点商贸物流设施建成投用，西部煤炭物流配送中心、宝鸡汽车博览园、育才机电汽车交易中心等项目开工建设。金融支持经济发展的能力不断增强，秦宝牧业上市通过证监会审核，瑞科新材料进入上市辅导期；市投资集团10亿元、宝钛集团7亿元企业债券获批发行；注入中小企业信用担保公司资本金3000万元，安排贷款风险补偿资金1000万元；招商银行宝鸡分行、西安银行宝鸡分行、中邮证券宝鸡营业部、中信建投宝鸡营业部先后开业，金融机构新增贷款113.26亿元，增长21.8%，居全省第二；新增小额贷款公司4户，总数达到11户，全市社会融资规模达到169.82亿元。保险业继续保持平稳运行，政策性农业保险覆盖领域逐步扩大，农业风险保障能力进一步提升。

城市服务功能不断增强，人居环境日益优越。“东扩南移北上”战略深入实施，城市规模不断扩张，建成区面积达到97.78平方公里，城镇化率达到45.61%，比上年提高2.57个百分点。南客站片区建设进展顺利，蟠龙新区道路建设不断加快，实现了供水、供气上塬。代家湾、姜城村等旧城改造项目加快推进，农贸市场、公共卫生间、绿地广场、停车场等50个市政设施项目全面完成，群众买菜难、如厕难、健身难、停车难等问题得到有效缓解；经二路综合改造提升、滨河南路、公园路立交桥等工程顺利竣工，新购置公交车95辆，新增公交线路3条，群众出行更加方便；姜谭、福临堡、金陵河、金台大道、陈仓中路等片区排水工程建成投用，市区内涝整治成效明显；群众路、姜城堡、神农片区集中供热工程全面完成，新增供热面积100万平方米，天然气利用三期工程进展顺利，惠及居民2.7万户；9个县城建设全面提速，建成区面积达到了42.43平方公里，新增3.62平方公里；４个省级重点示范镇的71个基础设施项目完成年度建设任务，投资达到21.59亿元。城市管理水平不断提升，国家卫生城市通过省级复审,国家卫生县城实现全覆盖，凤翔县在全省首家实现了数字化城市管理。国家生态园林城市创建深入开展，市区新增绿化面积279.9万平方米，安装了城市主干道隔离栏，建设了一批文化墙，对城市核心区及重要节点实施了整体亮化，渭河石鼓山坝桥结合工程、8.4公里的“一河两岸”改造提升工程建成投用，初步形成了集休闲、健身于一体的环湖景观。国家生态市创建工作全面启动，万元生产总值能耗下降3.6%，市区空气质量二级及以上天数达到315天，人居环境优势进一步彰显。

民生保障水平大幅提升，社会保持和谐稳定。坚持把保障和改善民生作为政府工作的出发点和落脚点，着力解决群众最现实、最迫切的民生问题。全年民生项目总投资达76.6亿元，为历年之最。加大就业创业扶持力度，技能培训22.8万人，发放小额担保贷款13.67亿元，扶持创业1.2万人，全市城镇新增就业5.8万人，城镇登记失业率控制在3.26%，农村劳动力转移就业106万人次，举办了第三届中国?宝鸡创业论坛，创业就业工作受到国务院表彰，荣获全国创业先进城市，市人社局被评为“全国就业先进工作单位”。社会保障扩面提标工作持续推进，3万名未参保城镇集体企业人员纳入企业职工养老保险，城乡居民养老保险参保人数达到150.28万人，城镇居民医疗保险、新型农村合作医疗参保率分别达到96.3%和98.95%，比上年提高1.3和1个百分点。四级社会公共服务平台基本建成，累计发行社会保障卡109万张，城乡群众享受到了更加方便快捷的公共服务。率先在全省建立了城乡居民家庭经济收入比对平台，城乡低保动态管理机制更加完善。全市投入6709万元，为残疾人办好十件实事。扩大了高龄老人生活保健补贴范围，提高了标准，22.1万名老人受益，养老机构床位达到7600张。开工建设保障性住房45708套，竣工26935套，有效缓解了中低收入家庭住房困难。双拥工作扎实开展，蝉联全国双拥模范城“七连冠”。坚持教育优先发展，全年教育支出达到53.9亿元，占公共财政支出的22.4%；实施“农村义务教育学生营养改善计划”和“蛋奶工程”，发放各类教育补助资金5.5亿元，惠及学生115万人次；加快实施学前教育三年行动计划，新建、改扩建公办幼儿园49所；建成省级标准化高中和示范高中各2所，市教育中心开工建设，市特殊教育学校基本建成；义务教育提升工程取得新进展，教育创强工作走在全省前列，市教育局荣获全国“两基”工作先进单位，受到国务院表彰。从解决群众“看病难”、“看病贵”问题入手，进一步深化市县两级公立医院改革，开展县级公立医院药品“三统一”试点，实行零差率销售，让利群众3700万元。城镇居民医保、新农合实现门诊统筹，大病统筹救助50641人，补助资金1.3亿元。市级医疗卫生信息平台、市食品药品检验检测中心、高新人民医院建成投用，荣获国家中医药工作、全国爱国卫生、全国无偿献血先进市，成为2012年全国医改最具影响力城市，荣登“2012中国医改政府支持榜”榜首。扎实推进全国人口计生综合改革示范市、幸福家庭试点市创建工作，开展了全国全员人口信息采集机制创新试点，累计发放奖扶资金1.18亿元，惠及群众20.6万人。国家公共文化服务体系示范区创建工作取得明显成效，市广电中心大楼顺利封顶，农家书屋、文化资源共享工程实现全覆盖，群众文化生活丰富多彩。全国全民健身示范城市创建工作成效显著，市游泳跳水馆开工建设，高质量承办了7项全国体育赛事和8项省十五运会年度比赛。认真落实信访工作“四项制度”，圆满完成了“一控两降”任务。“六五”普法全面启动，社会治安防控体系建设强力推进，严打整治成效显著，安全生产和应急管理不断加强，全市交通事故死亡人数、命案发案起数大幅下降，刑事发案率降幅达到40%以上，居全省第一。群众对社会治安满意度达到92.22%，比上年提高2个百分点，连续三年位居全省前列。

同时，妇女儿童、精神文明、民族宗教、国家安全、红十字会，以及监察、国土、审计、统计、档案、地志、粮食、烟草、供销、物价、地震、人防、气象、慈善等工作也都取得了新的成绩。

各位代表，过去的一年，面对宏观经济复杂多变、经济下行压力加大的严峻形势，我们始终坚持主攻招商引资、聚焦项目建设的鲜明工作导向，通过跑部进省向上争、千方百计从外引、发挥优势向内挖，着力解决项目小、项目少的问题，招商引资额、争取项目资金额均创历史新高，发展速度高于全省平均水平2.2个百分点，实现了关天副中心城市建设重点突破、加力提速；面对国家银根紧缩、调控加大的不利影响，我们始终坚持把金融支持实体经济作为稳增长、促发展的关键举措，通过不断强化金融服务和创新，深入开展“双推双增”融资工程，着力解决项目资金缺、企业融资难的问题，金融机构新增贷款和企业直接融资额均创历史新高，实现了金融对经济发展的强力支撑、良性互动；面对市强县弱、发展不平衡的短板制约，我们始终坚持全域规划、“四化同步”的发展思路，通过狠抓园区发展、城镇建设、现代农业，着力解决县域经济活力不足、特色不显的问题，县域经济占全市经济的比重达到47.6%，比上年提高1.2个百分点，实现了县域之间奋勇争先、竞相发展；面对打造务实、责任、服务、法治、诚信、廉洁政府的庄严承诺，我们始终坚持发展依靠人民、发展为了人民的执政理念，通过转作风、抓落实、求实效，着力解决干部作风不实、责任心不强等问题，全市各级干部干事创业的风气日益浓厚，人民群众对政府工作综合满意度达到85.31%，比上年提升2.2个百分点，对干部作风的满意度达到77.28%，比上年提升3.7个百分点。全市上下呈现出经济快速发展、社会和谐安宁、干群同心同力、人民安居乐业的可喜局面，宝鸡正朝着科学发展、加快发展、和谐发展的目标迈进。

这些成绩的取得，是市委总揽全局、正确领导的结果，是市人大、市政协鼎力支持、共谋发展的结果，是全市人民同心同德、顽强拼搏的结果。在此，我代表市政府，向人大代表、政协委员、各民主党派、工商联、各人民团体、社会各界人士，向驻宝企事业单位、人民解放军、武警驻宝部队全体官兵、公安干警，表示崇高的敬意和诚挚的感谢！

在充分肯定成绩的同时，我们也要正视发展中面临的困难和问题，主要是：经济总量不大，2012年仅占全省的9.76%，距离省上要求12%的目标还有较大差距，人均GDP的全面小康实现程度不足80%，全面建成小康社会任务繁重；经济结构不尽合理，民营经济占比较低，第二产业带动力不强，第三产业发展滞后，经济外向度不高，统筹城乡发展难度仍然很大；经济竞争力不强，创新能力有待进一步提升，在全国叫得响、有影响的品牌偏少；基础设施对整个经济社会发展的支撑作用不强，建设快捷交通体系、巩固提升交通枢纽地位的任务艰巨；尽管去年全市干部作风明显改进，群众满意度有了较大提高，但与建设关天副中心城市的新使命、广大群众的新期盼相比，还有一定差距。对于这些问题，我们将高度重视，采取有力措施，切实加以解决，绝不辜负全市人民的期望和重托。

2013年政府工作

今年，是深入贯彻落实党的十八大精神的开局之年，也是打造关天副中心城市的关键一年。当前，全球经济复苏仍有不确定因素，国内经济增长面临多重压力，宝鸡经济社会发展的外部环境依然复杂多变。但是，我们应当看到，宝鸡加快发展也面临新的机遇。中央将继续实施积极的财政政策和稳健的货币政策，从扩内需、调结构、促改革、惠民生和推进城镇化等方面加大对地方的投入；随着新一轮西部大开发、关中—天水经济区发展规划和省政府支持宝鸡加快建设关天经济区副中心城市意见的深入实施，中省将从产业开发、基础设施建设、生态环境治理等方面对宝鸡给予重点支持；国家六盘山片区、秦巴山片区区域发展与扶贫攻坚规划的启动实施，将极大地促进我市贫困地区加快发展和全面小康社会建设；省委、省政府在推进城镇化中把我市和西安作为构建全省城镇体系的第一层次，将出台一系列政策措施。可以说，宝鸡的发展有机遇、有基础、有条件，已经积蓄了强大的势能，处于加速崛起、跨越赶超的关键阶段。我们一定要准确判断形势，把握工作全局，保持清醒头脑，以强烈的使命意识、忧患意识，高点定位，换挡提速，自加压力，负重奋进，努力把宝鸡经济社会发展推向前进，为全面建成小康社会奠定坚实基础。

2013年政府工作的总体要求是：认真贯彻落实党的十八大精神，按照市委十一届三次全会确定的奋斗目标，强力推进项目建设，持续提升投资拉动作用；强力推进产业聚集，持续提升工业竞争优势；强力推进商贸旅游，持续提升三产贡献份额；强力推进现代农业，持续提升农民收入；强力推进基础设施建设，持续提升城镇辐射带动能力；强力推进生态文明建设，持续提升环境吸引力；强力推进民生保障，持续提升社会建设水平；强力推进作风转变，持续提升工作效能，打造关天副中心、建设和谐新宝鸡，推动“六个走在前列”迈出坚实步伐。

主要预期目标是：生产总值增长14.5%；全社会固定资产投资增长30%；地方财政收入同口径增长16%；规模以上工业增加值增长20%；城镇登记失业率控制在4%以内；城乡居民人均可支配收入分别增长14%、15%；物价涨幅控制在4%以内；人口自然增长率控制在5‰以内；万元GDP能耗下降3.5%，主要污染物排放量完成省定目标。

重点抓好以下六个方面的工作：

一、加速产业转型升级，持续增强经济实力

以扩大投资促总量、优化结构提效益、发展三产增活力为重点，推进产业升级，提高发展质量，强化对经济发展的支撑。

（一）加快重大项目建设，推动投资快速增长

强力推进招商引资。进一步扩大开放，坚持招大选优引强，持续支持中省企业技改扩能，增强发展后劲。深入挖掘自身优势和资源，以最好的产业、最好的项目、最好的资源对接战略投资者，引进世界或中国500强、行业100强企业20户。各县区、各园区主要领导要带头抓招商，亲自与客商洽谈项目，加大签约项目跟踪落实，坚持执行政策零折扣、优质服务零距离、环境保障零干扰的工作机制，促进重大项目落地，提高合同履约率、资金到位率和项目开工率，确保招商引资到位资金500亿元以上，增速达到15%。

加快建设重大项目。坚持一切围绕项目、一切为了项目，对重大项目优先配置土地、资金和环境容量。今年，全市安排重点建设项目300个，总投资2100亿元，年度投资510亿元，带动全社会固定资产投资超过1700亿元。突出抓好重大产业项目，加速推进海螺型材、关山风电、龙源风电、千阳钛产业工业园等项目建设，年内完成中铁宝桥扩能改造、青啤宝鸡工业园一期、农夫山泉二期等项目。创新重点项目管理，开辟绿色审批通道，建立项目保障机制；落实领导包抓责任，推行检查观摩、考核奖励制度，建立项目推进机制；严格稽查管理，对占地不建设或占有资源不开发超过期限的，收回土地和资源开发权，建立项目动态管理机制。做好重大项目前期工作，积极创造条件，确保一批成熟项目尽早开工建设。

积极争取项目资金。抓住国家扩大内需的政策机遇，紧跟国家产业政策和投资方向，围绕结构调整、产业升级、城镇建设、文化旅游、商贸物流，超前谋划，抓紧储备一批重大项目。继续加大中省资金争取力度，围绕社会事业、民生工程、生态环保、基础设施等方面，主动对接，争取更多项目进入中省计划盘子，确保项目总量增加、质量提高、接替有序。年内争取中省资金不少于150亿元，增幅达到15%以上。

（二）着力推进产业聚集，强化工业支柱地位

提升园区发展水平。优化园区产业布局，大力推进工业向园区集中。高新区以打造高新技术和高端装备制造业聚集区为重点，全力推进航天航空装备产业园、石油装备配套园、凌云蓄电池、机车基地等重点项目建设，生产总值增速达到20%以上。蔡家坡经开区坚持基础设施建设与招商引资并重，建成央企配套园和中小企业园，确保陕汽通力3万辆工程车、京泰1.5万吨高品质棉纱项目竣工投产，年内升格为国家级开发区。各工业园区要进一步明确产业定位，完善基础设施，加快标准化厂房建设，实施50个基础设施和产业项目，努力成为县域经济突破发展的主要承载地。实现渭滨姜谭、凤翔长青、凤州科技3个工业集中区产值过百亿元，扶风绛帐、眉县科技2个工业集中区过50亿元，确保县域工业集中区工业总产值增长22%。

做大做强优势产业。围绕打造“宝鸡?中国钛谷”，不断提升钛产业国际国内市场竞争力。以宝钛集团为龙头，拉长展宽加厚产业链条，促进企业之间合理分工、优势互补、共同发展，建设钛谷有色金属交易中心，启动国家级钛及钛合金检测检验分中心建设，钛产业产值年内突破350亿元。围绕打造百亿西凤，全面推进西凤酒城建设，壮大以西凤酒、太白酒、青岛纯生啤酒为骨干的食品产业。围绕麟北煤田开发，推进园子沟、招贤、李家河等井田建设，全年原煤产量达到1000万吨以上，加快宝麟铁路建设步伐，完成150万吨煤制甲醇一期工程，推进原煤就地转化。围绕高端装备制造业发展，尽快做大做强石油装备、航空电子、轨道交通等骨干产业。围绕九大产业集群发展，支持大企业大集团战略重组、技改扩张，发展壮大梯次跃进的大企业团队，新增产值或销售收入过百亿的企业1户、过50亿元的企业2户、过30亿元企业2户。

加快民营经济发展。认真落实国务院鼓励引导民间投资的“新36条”，激活民间投资。加大双向扶持政策力度，实施中小企业成长培育工程，增加中小企业贷款担保公司资本金，设立1000万元市级小微企业发展基金，对成长型小微工业企业的新增贷款给予贴息，建立中小企业服务体系，开通市级公共服务平台，带动全市工业关联发展、集约发展。培育壮大126户重点配套企业，重点培育104户小微企业，新增规模以上企业50户。

提高科技创新能力。加快国家创新型城市建设，支持秦川发展建设国家级工程技术研究中心，鼓励中铁宝桥、宝成航空、烽火电子等企业争创国家级企业技术中心。加速“两化”、军民融合，倾力打造公共科技创新服务、企业技术创新、产学研合作“三大平台”，加强与清华大学、北京大学、西北农林科技大学等省内外高校的校地、校企合作。加快国家科技成果转化示范基地建设，确保市科技创新服务中心建成投用，争创国家级文化科技融合示范基地。认真落实“三秦学者”、“百人计划”，积极引进各类高层次人才，为建设关天副中心城市提供人才支撑和智力保障。

大力发展建筑业。采取扶持奖励措施，引导中小型建筑企业在自愿的基础上，通过合并重组等形式，提升企业资质等级和市场竞争能力。放手发展民营建筑企业，支持本地企业开拓外埠市场，鼓励引导外地建筑企业、施工安装企业落户宝鸡。新增资质以上建筑企业15户，建筑业产值增长15%以上。

深化经济领域改革。积极推动国有企业改革，加快北照公司等3户企业政策性破产，做好陕铜公司等困难企业改制脱困工作，完成渭河工模具总厂与省电子信息集团重组。完善园区管理体制改革，赋予园区更大的自主权，推动园区快速发展。深化金融改革创新，鼓励商业银行和股份制银行在县区设立分支机构，实现小额贷款公司县区全覆盖。

（三）做大做强商贸旅游，提升三产支撑能力

加速发展旅游产业。编制完成宝鸡市文化旅游产业发展规划。加快法门寺文化景区开发，确保创建国家5A级景区一举成功。推进太白山国家级旅游产业新区建设,开展国家5A级景区创建工作,确保年内景区重新开放。完成茵香河两岸一期生态治理、石鼓文化城一期项目建设，打造周秦文化知名景区。启动金台观二期项目，加快凤县国家级羌族文化生态保护试验区建设，支持太白县建设西部“慢城”，推动扶风旅游示范县创建工作。开展特色旅游名镇、乡村旅游示范村、工业旅游示范点建设，进一步增强旅游吸引力。针对市场需求，设计精品旅游线路，加大宣传促销，优化旅游环境，吸引更多的游客进入宝鸡。新发展旅游企业20户，全年游客接待量和旅游综合收入均增长30%。

壮大商贸物流业规模。优化商业网点布局，抓好红星美凯龙、巨一时代广场、海航国际广场等重大商贸项目建设，发展限额以上商贸企业200户。扶持供销企业做大做强，构建农业生产资料、日用消费品、农副产品、再生资源四大现代流通网络，建成西部丰茂再生资源综合利用产业基地。加快陈仓物流园区新港物流中心、汽车博览园等项目建设，打造投资洼地，吸引大型物流企业向园区聚集。组建专门机构，编制相关规划，推动陆港新区前期工作。设立服务业发展引导资金，抓好传统服务业改造升级，大力发展工业设计、技术研发等生产性服务业，积极发展中介、创意、服务外包等新兴服务业。实施龙头企业培育、外贸企业孵化、出口基地创建、国际市场开拓“四大工程”，努力扩大进出口规模，全年实现外贸进出口总额8.2亿美元。

积极促进城乡消费。大力推进“万村千乡市场工程”、“镇超工程”和“农超对接工程”,健全流通体系,优化消费环境。推行消费信贷,引导汽车、住房等大宗消费，促进家政服务、文化休闲消费。大力发展“银发消费”,满足老年人口的消费需求。落实带薪年休假制度，鼓励旅游、文体等消费。组织引导商贸流通企业，开展集中促销活动，促进餐饮、服装、家电等消费。

加快金融业发展。稳妥发展融资性担保机构，新引进各类金融机构3户以上，新增小额贷款公司、融资性担保公司5户以上。引导企业开展直接融资，做好西凤酒业、瑞科新材料、力兴钛业、赛孚石油、海浪锅炉等企业上市的前期工作，年内完成红旗民爆首发上市。积极发行新材料高新技术创投基金，规范运作高端装备制造高新技术创投基金、宝鸡红土创投基金，扩大对产业和项目的投资。年内力争高新开发总公司企业债券、秦川发展公司债券及东岭集团、烽火集团短期融资券获批发行。优化金融生态环境，新增各项贷款110亿元，社会融资规模达到160亿元以上。

二、高度重视“三农”工作，促进农民持续增收

大力发展现代农业。着力提升宝鸡国家级休闲农业示范区、15个省级现代农业园区建设水平，新创建省级园区5个。深入开展粮食高产创建活动，抓好扶风、凤翔等6个国家级和省级产粮大县建设，实施“放心粮油全覆盖”工程。抓好眉县、陇县、千阳县3个千头奶牛场以及陈仓坪头、岐山南阳等10个万头生猪养殖示范村建设，加快新希望、山东大成、石羊集团肉鸡产业化项目进度，提升畜牧业规模化水平。按照部省共建的原则，加快眉县国家级猕猴桃批发交易中心建设，做大做强猕猴桃产业，使猕猴桃成为宝鸡农产品进军国际市场的领头雁；加快建设渭北塬区苹果、南山西山花椒、关山乔山核桃产业带，建成果业万亩区3个、千亩方10个，促进果业扩面提质增效。大力发展蔬菜生产，突出抓好川塬县区设施蔬菜基地建设，集中连片开发一批千亩蔬菜园；积极发展太白、凤县等山区县绿色无公害蔬菜，提高蔬菜生产效益。稳步推进农村产权制度改革，引导农村集体土地依法、规范、有序流转，支持龙头企业、专业大户、农民专业合作社和家庭农场发展，提高农业产业化程度，新增市级以上龙头企业10户。

改善农业农村基础条件。建设渭河堤防22公里、支流入渭口桥梁7座，实现渭河北岸堤防全线贯通，建设渭河北岸70公里芦苇及水生植物绿色生态景观长廊，确保治渭工作继续走在全省前列。实施高标准基本农田建设、小流域综合治理等重点工程，建设基本农田4万亩，发展节水灌溉10万亩，治理水土流失200平方公里，完成天堂河等4条中小河流治理任务。加快建设农村人饮工程，解决全市13万农村人口安全饮水问题，抓好岐山全国农村饮水示范县和麟游省级整体推进县建设。做好段家峡水库渭北灌区、普化水库等重大项目前期工作，不断提高全市水资源持续供给能力。深入实施“晋星强基”、“统筹提升”工程，建成五星村30个、四星村100个，支持高新区、蔡家坡、凤县建设统筹城乡发展示范区。

持续增加农民收入。依托主导产业，加大实用技术培训力度，重点抓好眉县、千阳高级奶农培训中心建设，全年开展农民科技文化教育100万人次，培育职业农民2000人，依靠科技创新和提高劳动者素质，增加农民经营性收入。优化就业信息服务，促进农村劳动力转移就业，年内转移农村劳动力100万人以上，增加农民工资性收入。搞好承包地、林地、宅基地和房屋产权的确权颁证试点工作，增加农民财产性收入。全面落实各项强农惠农政策，积极推进城乡公共服务均等化，增加农民转移性收入。

加大扶贫开发力度。以国家级两个片区扶贫规划实施为契机，深入持续突破西山，加快推进北部乔山、西部关山、南部秦岭三大区域十三个板块扶贫工作，继续抓好基础设施改善、主导产业培育、扶贫移民搬迁、信贷资金扶持等重点工作。启动86个村整村推进项目、8个村世行扶贫项目，扶贫移民搬迁8000人，加大行业和社会扶贫力度，落实社会帮扶资金2500万元以上，年内实现7.5万人脱贫。

三、积极推进城镇化，提高辐射带动能力

构建快捷交通体系。高速公路以构建“十字加外环”主骨架为重点，加快建设连霍高速市区过境段、宝汉高速、绛法高速。省级干线公路以提升通行能力为重点，完成西宝北线扶风至凤翔段、西宝中线陈仓段改扩建，加速推进212省道凤县、陇县县城过境段、306省道崔木至麟游县城段、眉麟公路岐山段等公路改建项目。以实施列入国家集中连片特困地区交通建设项目为重点，改善贫困地区交通条件。启动宝鸡支线机场、210省道麟凤段升等改造、凤翔大道、关中环线眉县汤峪至岐山段一级公路等前期工作。加快宝兰客专建设步伐，确保西宝客专建成试车，巩固强化西部交通枢纽地位。

加快城市新区建设。按照“提升标准、加快建设”的要求，加速推进城市“东扩南移北上”。实施虢镇东门十字综合体等项目，开工建设陈仓区北新路一期工程，拓宽改造磻蔡路，完成陈仓区集中供热二期工程、渭河北岸排水工程建设；加快科技新城建设步伐，积极推进南客站站房、站前广场、站前大道及地下工程建设，开工建设村民安置楼；加快推进蟠龙新区建设，完成蟠龙大道、代家湾上塬路、上塬供水项目，开工建设蟠龙新区主干道路、给排水、供电、供热、供气、污水处理厂等一批项目，启动建设金台区行政中心，做好斗中路到蟠龙新区交通建设前期工作。

增强城市载体功能。按照“完善功能、提升形象”的要求，完善老城区服务功能。全面完成181个旧城区基础设施改善提升项目建设任务，新建一批市场、停车场、小街巷、街头绿地、公厕、垃圾转运站等设施。加快新东岭、代家湾盛世广场、世纪荟萃广场、南关路等十大城市综合体建设，积极实施光明村、姜城村、郭家崖村等城中村改造项目。完成石头河引水一期工程，实现向市区供水。新增集中供热面积100万平方米，完成供热分户计量改造150万平方米。新增天然气用户2.6万户，启动代家湾储气站搬迁扩容项目。大力发展公共交通，调整优化公交线路，建设城市公共自行车系统，方便群众出行。

完善城镇基础设施。以凤翔撤县设区、蔡家坡市政设施完善、陇县南岸新城建设为重点，强力推进三个副中心建设。加大县城基础设施建设力度，扩张规模，提升品位，增强承载能力。加快4个省级重点示范镇建设，年内完成投资14亿元。提高其它建制镇建设水平，增强公共服务功能，有序推进农业转移人口市民化。

提高城市管理水平。严格规划管理，建立城市总体建设风貌控制体系。加快智慧城市建设，不断提升数字化城管运行水平。强化城市综合执法，深入开展市容市貌、交通秩序、市场秩序整治，加强户外广告、渣土车、城市养犬管理，提升城市形象。抓好城市常态化环卫保洁工作，打造西部最干净城市。加强物业市场管理，建立投诉受理机制，保障业主合法权益。制定房地产开发企业诚信管理制度，促进房地产市场健康发展。

四、持续改善生态环境，全力建设美丽宝鸡

抓好绿化美化。深入推进“绿色宝鸡”九大工程，抓好千里绿色长廊、渭河北坡、陕西西大门等重点区域绿化，开展森林围城、围镇、围村建设，实施退耕还林后续产业、千山重点区域绿化等项目，完成工程造林25万亩，义务植树800万株，实现城镇绿化提升、农村绿化覆盖，率先推进关中大地园林化。加快千渭之会国家湿地公园试点建设，推进森林进企业、进矿区、进校园、进社区、进单位，积极创建国家生态园林城市，让宝鸡大地绿意盎然、生机勃勃。

加强节能减排。加大落后产能淘汰关停和技术改造力度，抓好锅炉改造、余热余压利用等工程，建成宝二电二号、三号机组、冀东水泥等7条水泥生产线的烟气脱硝设施。实施产业园区改造等循环经济项目，建设“城市矿产”示范基地。严格执行新的排污标准，全面降低污染排放总量，确保完成年度节能减排任务，通过国家环保模范城市复检验收。继续抓好节水型社会试点，充分利用地热、中水、太阳能等资源，大幅降低能源、水、土地消耗强度，给自然留下更多的修复空间。从低碳办公、绿色生活做起，真正将节约节能理念转变为居民的自觉行动。

保护生态环境。努力建设天蓝地绿水净的美丽宝鸡。实施关中地区大气联防联控方案，启动空气质量PM2.5监测，抓好车辆尾气、餐饮油烟、建筑工地、医疗废弃物等治理，建立城市环境管理长效机制。继续开展渭河治污三年行动，推进小韦河、千河流域环境综合治理，严格实行渭河流域县区断面水质监测考核，做好麟北煤田、非煤矿山的生态环境保护工作。深入开展国家生态市、生态县创建活动，太白、千阳通过省级生态县技术评估，建成一批生态镇和生态村。持续推进农村环境集中连片综合整治，完成88个村的示范项目。建设长寿沟循环经济示范园区，建成市餐厨垃圾处理厂、城肥处理厂。提标改造十里铺污水处理厂，建成岐山县城污水处理厂，确保建成的污水处理厂正常运行，坚决杜绝垃圾处理场二次污染。

五、大力发展社会事业，提高公共服务水平

办好人民满意教育。深入推进“教育振兴八大工程”，优化学校规划布局，不断满足群众教育需求。实施薄弱学校改造等项目，大力改善办学条件。加快发展学前教育，全年新建、改建乡镇中心幼儿园和城区幼儿园35所，实现公办乡镇中心幼儿园全覆盖。大力推进标准化、示范性学校建设，创建省级标准化高中和示范幼儿园各2所。继续推进“双高双普”和教育创强工作，加快市教育中心、宝鸡高新一中建设，确保市特殊教育学校上半年开学。实施职业教育品牌建设工程，成立职业教育集团，宝鸡职业技术学院通过省级示范性高职院校验收。积极推进教育教学改革，加强教师全员培训，完善校长、教师双向交流制度，促进教育均衡发展。

提高医疗卫生水平。完善公共卫生服务体系，加强公立医院监管，提高服务能力。推进新农合市级统筹，开展大病保险试点，推行基本药物制度，启动全市第二次医疗服务价格调整。优化医疗服务机构布点，方便群众就医。加快市精神卫生中心、市口腔医院综合楼建设，建成市中心医院内科大楼、市第三人民医院综合楼。加快推进市县区域卫生信息平台建设，实现医疗信息资源共享。完善重大疾病、重点传染病防控机制，提高突发公共卫生事件应急处置能力。强化医德教育和名医培养，提升医疗卫生队伍服务水平。开展爱国卫生运动，巩固国家卫生城市创建成果，确保通过国家复审。加快市级人口家庭公共服务中心建设，持续开展优生促进、母亲健康等系列服务工程，扎实推进全国全员人口信息采集机制创新试点，全面提高人口素质。

丰富群众文化生活。发挥文化引领风尚、教育人民、服务社会、推动发展的作用，促进文化大发展大繁荣。强力推进全国文明城市创建，不断提高全社会文明素质。开工建设市科技馆、图书馆、青少年活动中心，启动建设3个省级文化产业示范基地。全力做好国家公共文化服务体系示范区创建工作，确保通过国家验收。积极实施文化惠民工程，加快“两馆一站”建设，全面落实文化馆（站）免费开放政策。深入开展“书香陈仓”读书月和广场文化、社区文化等活动，丰富城乡群众文化生活。加强非物质文化遗产保护，办好中国首届社火艺术节。加大扶持力度，培育一批文化骨干企业，推动文化产业发展。确保市广播电视中心竣工投用，实施好广播电视“户户通”工程，实现农村广播电视公共服务全覆盖。做好北首岭、雍城、周原、九成宫等大遗址的保护、开发和利用，在国家博物馆举办“守望家园”—陕西宝鸡群众保护文物特别展览。做好省十五运会的宣传、筹备工作，建成市游泳跳水馆、射击射箭馆，完成市体育场、馆改造提升工程，办好市第十届运动会。深入开展全民健身月活动，建成全国全民健身示范城市。

用好管好财政资金。不断壮大骨干财源，大力培育新兴财源，切实增强财政实力。深化部门预算改革，今年部门预算公开单位扩大到60家。切实加强财政专项资金管理，积极推进预算绩效评价改革，对200万元以上的民生工程、基础设施、转移支付等项目实施绩效评价，提升财政资金使用效益。优化财政支出结构，确保新增财力的80%以上用于保障和改善民生。

六、不断强化民生保障，促进社会和谐稳定

全面推进创业就业。深入推进就业优先战略，整合并轨各类扶持政策，做好供需对接、就业信息等相关服务工作，扩大“宝鸡技工”品牌影响力，创业就业培训4万人，新增城镇就业5万人。积极实施创业带动战略，加快创业就业服务基地建设，发放小额担保贷款10亿元，打造西部创业论坛品牌，巩固提升国家创业型城市成果。

提升社会保障水平。完善“大社保”宝鸡模式，促进“解困民生”向“普惠民生”转变。认真落实中省市已出台的各项政策，推进提标扩面，提高社会保障水平。加快社会保障卡建设工程，使150万群众能够享受便捷的社会保障服务。健全居民家庭经济状况比对工作长效机制，切实做到应保尽保。加快市综合减灾与公共安全科教培训中心、军休管理服务中心、绛帐社会福利院迁建项目建设，做好零散烈士纪念设施抢救保护工作。加大对老年事业的投入，鼓励更多的民间资本投入养老事业，在渭滨区建成全市首个社区老年人服务信息平台。继续为全市残疾人办好十件实事。

切实抓好住房保障。加快虢镇茗苑、龙山雅居、金河尚居等市区保障性住房小区建设，抓好各县保障性住房项目，年内建成保障性住房2.67万套以上。加强项目建设管理，确保工程质量，搞好保障房的配套设施建设，把保障房分配到符合条件的群众手中。深入实施农村危房改造项目，建设“三无户”安全住房1000户，切实解决农村居民家庭住房困难。

推进社会管理创新。创新社区管理体制和运行机制，加快4个城镇社区服务站、30个农村社区服务中心建设，完善167个农村社区服务中心配套设施，启动第五次城镇社区居民委员会换届选举工作。扎实推进平安宝鸡建设，健全网格化、立体化、智能化治安防控体系，保持对各类违法犯罪的高压态势。健全大调解机制，畅通信访渠道，注重源头治理，妥善解决群众合理诉求。进一步强化生产安全、交通安全、校园安全、消防安全的监管整治，有效预防重特大事故发生。加大食品药品监管力度，整合检测资源，确保群众饮食用药安全。推广渭滨区石鼓镇流动人口管理经验，切实做好特殊人群、网络虚拟社会管理。健全应急管理和应急救援体系，提高预防自然灾害和应对突发事件的能力。

加强政府自身建设

各位代表，全面完成今年的各项目标任务，市政府肩负着重任，承载着厚望。我们将不断加强自身建设，忠实履行职责，树立“为民、务实、勤政、清廉”的良好形象，努力建设人民满意的服务型政府。

加强学习，深入调研，提高政府决策力。认真学习党的十八大精神，营造崇尚学习的良好风气。各级政府组成人员要时刻把群众的安危冷暖放在心上，多想想困难群众，多想想贫困地区，深入基层，深入群众，蹲点驻村进企调研，多渠道了解社情民意，从群众中汲取智慧和经验，使决策更加贴近群众意愿。实施重大事项科学决策制度，坚持专家咨询、公众参与、社会公示、集体讨论等决策程序，充分发挥市决策咨询委员会作用，努力提高科学决策、民主决策、依法决策水平。真正使我们做出的决策、所干的工作，既向上级负责、又向群众负责，既向现实负责、又向历史负责。

解放思想，与时俱进，提高政府创新力。树立全球化视野、国际化眼光、现代化标准，以“跳起来摘桃子”的精神和勇气，把各项工作放在全国、全省大格局中找差距、寻位置、定目标，瞄准先进、奋力赶超、争创一流，不断创造新的业绩。把改革作为最大的红利，破除因循守旧观念，增强改革创新意识，用创新的办法破解产业结构调整、经济转型升级、项目资金制约、维护社会稳定等发展中的难题。鼓励创新，宽容失败，激发创造活力。

转变作风，狠抓落实，提高政府执行力。认真落实中央和省、市委进一步改进工作作风、密切联系群众的有关规定，深入开展以为民务实清廉为主要内容的群众路线教育活动。下基层要坚持轻车简从、减少陪同、简化接待，力戒走过场和形式主义。切实改进文风会风，提倡少开会、开短会，少发文、发短文，严格控制以政府名义召开全市性会议和举办大型活动，加快电子政务建设，提高办文、办会、办事效率。把抓落实作为政府工作的永恒主题，安排部署工作，能量化的要尽可能量化，不能量化的要界定清楚，切实做到目标任务、措施办法、时间进度、责任人员、成效结果“五明确”。要敢啃硬骨头，敢抓关键点，忠诚履责，尽心负责，勇于担责。进一步改进工作方式，简化手续，特事特办，急事先办，要事快办，做到定了就办、说了就干，确保令行禁止，政令畅通。加大督办考核力度，强化效能监察，提高政府工作的执行实效。

依法行政，讲求诚信，提高政府公信力。各级政府及其组成部门要树立依法行政理念，按照法定权限和程序行使权力、履行职责，着力推进法治政府建设。继续开展创建全省依法行政示范市活动，规范行政处罚自由裁量权工作，不断提高依法行政水平。深化行政审批制度改革，精简行政审批环节。强化政务公开，继续加大食品安全、征地拆迁等重点领域的信息公开，增强政府工作的透明度和公信力，保障人民群众的知情权、参与权、表达权、监督权。自觉接受人大及其常委会的依法监督、政协的民主监督、新闻舆论和人民群众的公开监督，虚心听取社会各界的意见、建议，认真办理人大代表议案、建议和政协委员提案。

廉洁奉公，执政为民，提高政府凝聚力。为政清廉才能取信于民，秉公用权才能赢得民心。继续深化预防腐败工作，注重用制度管权管事管人，加强对领导干部和重点领域、关键环节、重要岗位的监督，做到行政权力运行到哪里，监督就落实到哪里，财政资金运用到哪里，审计就跟进到哪里。严肃查办违法违纪案件，始终保持惩治腐败的高压态势。深入推进纠风专项治理，严肃整治消极懒惰、作风散漫、奢靡享乐等不良风气。牢固树立过紧日子的思想，压缩“三公”经费支出。全体公务人员要克己奉公、廉洁自律，切实做到政治清醒、为政清廉、为人清白。

各位代表，宝鸡的发展充满希望，宝鸡的前景无限美好。让我们在中共宝鸡市委的坚强领导下，以党的十八大精神为指导，紧紧依靠全市人民，凝聚力量，开拓创新，勇于实践，攻坚克难，为打造关天副中心、建设和谐新宝鸡而努力奋斗！
